# Would anti-choking devices be correctly and quickly managed by health science students? A manikin crossover trial

**DOI:** 10.1186/s12909-023-04345-7

**Published:** 2023-05-23

**Authors:** Borja Cardalda-Serantes, Aida Carballo-Fazanes, Emilio Rodríguez-Ruiz, Cristian Abelairas-Gómez, Antonio Rodríguez-Núñez

**Affiliations:** 1 Anesthesiology and Intensive Care Medicine Department, University Clinic Hospital of Santiago de Compostela (CHUS), Galician Public Health System (SERGAS), Santiago de Compostela, Spain; 2 CLINURSID Research Group, University of Santiago de Compostela, Santiago de Compostela, Spain; 3 Primary Care Interventions to Prevent Maternal and Child Chronic Diseases of Perinatal and Developmental Origin (RICORS), Instituto de Salud Carlos III, RD21/0012/0025 Madrid, Spain; 4 Simulation, Life Support, and Intensive Care Research Unit (SICRUS), Health Research Institute of Santiago de Compostela (IDIS), Santiago de Compostela, Spain; 5 Intensive Care Medicine Department, University Clinic Hospital of Santiago de Compostela (CHUS), Galician Public Health System (SERGAS), Santiago de Compostela, Spain; 6 Faculty of Education Sciences, Universidade de Santiago de Compostela, Santiago de Compostela, Spain; 7 Pediatric Critical, Intermediate and Palliative Care Section, Pediatric Department. Hospital, Clínico Universitario de Santiago de Compostela, Santiago de Compostela, Spain

**Keywords:** Airway clearance, FBAO, LifeVac®, DeCHOKER®, Nursing and medical students, Simulation

## Abstract

**Background:**

The brand-new anti-choking devices (LifeVac® and DeCHOKER®) have been recently developed to treat Foreign Body Airway Obstruction (FBAO). However, the scientific evidence around these devices that are available to the public is limited. Therefore, this study aimed to assess the ability to use the LifeVac® and DeCHOKER® devices in an adult FBAO simulated scenario, by untrained health science students.

**Methods:**

Forty-three health science students were asked to solve an FBAO event in three simulated scenarios: 1) using the LifeVac®, 2) using the DeCHOKER®, and 3) following the recommendations of the current FBAO protocol. A simulation-based assessment was used to analyze the correct compliance rate in the three scenarios based on the correct execution of the required steps, and the time it took to complete each one.

**Results:**

Participants achieved correct compliance rates between 80–100%, similar in both devices (*p* = 0.192). Overall test times were significantly shorter with LifeVac® than DeCHOKER® device (36.6 sec. [31.9–44.4] vs. 50.4 s [36.7–66.9], *p* < 0.001). Regarding the recommended protocol, a 50% correct compliance rate was obtained in those with prior training vs. 31.3% without training, (*p* = 0.002).

**Conclusions:**

Untrained health science students are able to quickly and adequately use the brand-new anti-choking devices but have more difficulties in applying the current recommended FBAO protocol.

## Background

Foreign body airway obstruction (FBAO) is a medical emergency that represents the fourth leading cause of potentially preventable and treatable accidental death both at home and in the community [1, 2]. Also, it has been reported as a leading cause of death in 1 to 3 aged kids, ahead of traffic accidents [3, 4]. Kids younger than 3 years old together with people over 65 years old and patients with musculoskeletal and neurologic conditions represent the main population at risk [3, 5].

In case of a FBAO event, early intervention by bystanders in out-of-hospital setting has been associated with a better neurological prognosis for the victim [2–6].

In this sense, scientific societies have urged to provide first aid training to parents, education professionals, kids, and elderly caregivers [5, 7]. In addition, previous studies suggest the need for adequate training in this field for laypeople, health science students, and healthcare professionals. The latter are also responsible of broadcast information about activities and other preventive measures to both their patients and their caregivers [7], therefore, seems essential that their training should be adequate.

The current recommended protocol to treat FBAO combines back blows and abdominal thrusts for resuscitation of a choking victim, progressing to cardiopulmonary resuscitation (CPR) if those manoeuvres are ineffective and the victim loses consciousness [1, 8]. Recently, anti-choking devices (LifeVac® and DeCHOKER®) have been developed to treat FBAO as a second step in the face of the ineffectiveness of standard manoeuvres [9–11]. Besides, these devices are widely available for general population use [12]. Currently, these externally applied, portable, non-powered, suction-generating devices are only registered as Class 1 FDA ‘suction apparatus’ [9, 13, 14]. There is limited high-quality scientific evidence about these devices to support or disapprove them [10, 13].

Considering the challenge to carry out high-quality research in this field [15], with the hypothesis that people without training are able to use the brand-new anti-choking devices, this study aimed to assess the ability of health sciences students (Medicine and Nursing) to manage LifeVac®, DeCHOKER® and the recommended protocol in a simulated adult FBAO scenario.

## Methods

### Aim, design and setting of the study

This manikin crossover trial study carried out at the University of Santiago de Compostela aimed to assess the ability to use the brand-new anti-choking devices (LifeVac® and DeCHOKER®) in an adult FBAO simulated scenario, by untrained health science students.

### Participants

A convenience sample of 43 health science students (nursing and medical students in any year of their degree) from the University of Santiago de Compostela without prior training in anti-choking devices took part in this study. Before the tests, all the participants signed an informed consent, explaining the study's aims, agreeing to give up their data for research purposes (always treated anonymously), and informing them that they could leave the study at any time. The study was conducted under the amended Declaration of Helsinki. The Research Ethics Committee of Santiago-Lugo did not consider it necessary to review the research protocol since it is a simulation study.

### Procedure

A manikin randomized crossover study was performed. Three FBAO events were simulated to each participant, who without prior training, was encouraged to resolve them with 1) LifeVac® device (LifeVac® test), 2) DeCHOKER® device (DeCHOKER® test), and 3) following the recommendations of the current protocol for action. The start of these scenarios was randomized for the participants using a random generator.

For assessing the anti-choking devices, an adult manikin (Little Anne QCPR™; Laerdal) was used as a simulated FBAO victim. Participants had to try to resolve it only with the help of the manufacturer’s leaflet instructions, which were provided on paper as they are in reality accompanying the corresponding anti-choking device. However, for the test which evaluates the recommended protocol, a real victim who simulated a FBAO event (first mild and finally severe obstruction) was used. Participants had no instructions beyond their knowledge to solve it. No training was performed before each test, nor was any information provided to them during the tests; letting them act as if they were alone in the FBAO scenario.

Data, related to the performance or non-performance and correct or incorrect executions of each step of the above-mentioned tests, were collected in a specific checklist by one researcher while another recorded the time spent on each step and the overall time test.

### Materials

Two anti-choking devices (LifeVac® and DeCHOKER®) and a manikin (Little Anne QCPR™; Laerdal) were used in our study.

LifeVac® device consists of a mask with a patented valve to create a seal [9, 13, 14]. The second component, connected to the first one through a one-way valve, consists of the plunger. This plunger, when compressed, will cause a unidirectional suction phenomenon that will try to dislodge the foreign body from the airway, preventing it from moving deeper into the airway [16] It includes 3 types of interchangeable masks: a small paediatric mask (children between 1–4 years old weighing more than 10 kg), a large paediatric mask (children over 4 years old), and one for adults [5, 17, 18].

DeCHOKER® device consists of a plunger-type system, responsible for generating the negative pressure and unidirectional suction current necessary to dislodge the foreign body (solid or liquid) and clear the airway. Unlike LifeVac®, it also has an oropharyngeal component, which simulates an oropharyngeal airway [13]. It is available in three different sizes: infants (between 1-5 years old), children (between 5-12 years old) and adults (from 12 years, including wheelchair patients and pregnant) [19].

Little Anne QCPR™ (Laerdal) mannequin was used as a simulated FBAO victim for the resolution of the two tests which evaluate both anti-choking devices.

### Variables

Characteristics of the participants (age, sex and undergraduate degree) were recorded. In addition, data on their knowledge and subjective assessment of their ability to act in the event of a FBAO situation were collected, as well as whether they had witnessed and/or acted in a FBAO event on some occasion and when.

The primary variables of this study were: the proper execution of each of the steps required in the handling of the anti-choking devices and in the recommended protocol (categorical variables) and the time (in seconds) it took to resolve the scenarios (continuous variable). The correct compliance rate (%) was calculated according to the next equation (Ʃ steps correctly performed × 100)/number of steps assessed).

LifeVac® correct compliance rate was calculated given correct or incorrect execution of the items of this sequence: 1) insert the mask on the device's bellows, 2) place the mask correctly covering the victim’s nose and mouth, 3) fix the mask to the victim's airway, 4) push in the handle/bellows, 5) pull the handle upwards, 6) keep the mask fixed to the victim’s airway throughout the procedure.

DeCHOKER® correct compliance rate was calculated by evaluating correct or incorrect execution of the items of this sequence: 1) place the device correctly, 2) fix the mask to the victim's airway, 3) pull the plunger out with force, 4) keep the mask fixed to the victim’s airway in place throughout the procedure.

The current recommended protocol of action correct compliance rate was calculated taking into account the correct or incorrect execution of the items of this sequence: 1) encourages coughing, 2) performs back blows, 3) correctly performs back blows, 4) performs abdominal thrusts, 5) correctly performs abdominal thrusts, 6) continues 5 back blows × 5 abdominal thrusts, 7) correctly continues 5 × 5, 8) indicates initiation of CPR manoeuvres in case of unconsciousness (1). These variables were compared between participants who had prior training in FBAO recommended protocol and participants who had no previous training.

Lastly, at the end of each test, participants were questioned on a subjective variable, the election of one of the two anti-choking devices (LifeVac® and DeCHOKER®).

### Statistical analysis

First, a descriptive analysis was performed. Categorical variables were expressed with frequencies and percentages. Continuous variables were expressed with median and interquartile range (IQR), according to their adjustment to a non-normal distribution (Shapiro–Wilk test). When comparing categorical variables, Chi-square statistic was performed, or Fisher’s Exact Test when the number of cells with expected values ≤ 5 was over 20%. Comparisons between LifeVac® and DeCHOKER® continuous variables were performed using the Wilcoxon signed-rank test and between participants with and without training in the recommended protocol with the Mann Whitney U test. Analysis was performed using the SPSS statistical software (IBM corp., v. 25.0 for Mac), and for all analyses, a *p*-value of less than 0.05 was statistically significant.

## Results

Overall, 43 health science students from the University of Santiago de Compostela participated in the study: 24 nursing (55.8%) and 19 medical students (44.2%). Characteristics of the participants are presented in Table 1. Thirty-one (72.1%) participants referred to had previous training on the current recommended protocol. Before the study 28 (65.1%) participants considered themselves capable of resolving an FBAO event. However, only 5/43 participants had witnessed and 3/5 of them had acted on a FBAO event (Table 1).

**Table 1:** Characteristics of the participants

**Variables**	**Participants ***n* = 43
**Age (years)**	21.0 (21.0 – 23.0)
**Sex**	
Male	27 (62.8)
Female	16 (37.2)
**Degree**	
Nursing	24 (55.8)
Medicine	19 (44.2)
**Prior training in FBAO**	
Yes	31 (72.1)
No	12 (27.9)
**Years since training**	3.0 (1.0 – 3.0)
**If you witness a FBAO, would you be able to solve it?**	
Yes	28 (65.1)
No	15 (34.9)
**Have you ever witnessed a FBAO?**	
Yes	5 (11.6)
No	38 (88.4)
**Years since having witnessed the FBAO**	8.0 (3.3 – 10.5)
**Have you intervened when the FBAO?** (*n* = 5)	
Yes	3 (60.0)
No	2 (40.0)

*kg* kilogram; *m* meters; *FBAO* Foreign Body Airway Obstruction

Continuous variables are expressed with median (interquartile range)

Categorical variables are expressed with absolute frequency (relative frequency)

A descriptive analysis of the participants’ performance with LifeVac® and DeCHOKER® anti-choking devices is shown in Table 2. Even though the median estimated correct compliance rate with both devices is 100%, only 62.8% of participants performed all steps correctly with the LifeVac® device *vs*. 81.4% with the DeCHOKER® device (*p* = 0.125). Although there were no significant differences, “to keep the mask fixed to the victim’s airway throughout the procedure” was the most failed step in both tests (74.5% with LifeVac® vs. 86.0% with DeCHOKER®; *p* = 0.164).

**Table 2:** Descriptive analysis of the participants’ performance with LifeVac® and DeCHOKER® devices during an adult victim FBAO

Variables	LifeVac®	DeCHOKER®	*p*-value
**Place the mask correctly covering the victim’s nose and mouth**			1.000
Yes	38 (88.4)	42 (97.7)	
No	5 (11.6)	1 (2.3)	
**Fix the mask to the victim’s airway**			0.007
Yes	39 (90.7)	41 (95.3)	
No	4 (9.3)	2 (4.7)	
**Push in handle**			-
Yes	42 (97.7)	-	
No	1 (2.3)	-	
**Pull handle (LifeVac®) // Pull the plunger out with force (DeCHOKER®)**			-
Yes	43 (100.0)	43 (100.0)	
No	0 (0.0)	0 (0.0)	
**Keep the mask fixed to the victim’s airway throughout the procedure**			0.164
Yes	32 (74.4)	34 (86.0)	
No	11 (25.6)	6 (14.0)	
**Perform all the steps correctly**			0.125
Yes	27 (62.8)	35 (81.4)	
No	16 (37.2)	8 (18.6)	
**Correct compliance rate**	100 (80.0 – 100.0)	100 (100.0 – 100.0)	0.192^a^

Continuous variables are expressed with median (interquartile range)

Categorical variables are expressed with absolute frequency (relative frequency)

*FBAO* Foreign Body Airway Obstruction

*p-values* calculated by Chi-square test or Fisher’s Exact Test as appropriate

^a^Wilcoxon test

Table 3 shows the comparative analysis of test times and estimated correct compliance rate between both anti-choking devices. Overall test times were significantly shorter when using LifeVac® compared with DeCHOKER® (36.6 s. [31.9 – 44.4] *vs*. 50.4 s. [36.7 – 66.9], *p* < 0.001). Participants achieved high and similar correct compliance rates with both devices.

**Table 3:** Comparison of procedure time and compliance rate between LifeVac® and DeCHOKER®

Variables	LifeVac®	DeCHOKER®	*p*-value
Time to device fitting on the victim	29.3 (25.7 – 37.5)	36.8 (26.2 – 49.2)	-
Total time	36.6 (31.9 – 44.4)	50.4 (36.7 – 66. 9)	< 0.001
Correct compliance rate	100 (80.0 – 100.0)	100 (100.0 –100.0)	0.192

Data are expressed as median (interquartile range)

*p*-values calculated by Wilcoxon test

Regarding the current recommended protocol performance (Table 4), none of the untrained participants performed all the steps, so the overall test time is significantly shorter compared with trained participants (42.7 *vs*. 56.5 sec., *p* = 0.002). The correct compliance rate is significantly lower in those participants without prior training (31.3% *vs*. 50%, *p* = 0.002).

**Table 4:** Descriptive analysis of the participants’ performance of current recommended steps to treat an adult victim with FBAO

**Variables**	**Overall** (*n* = 43)	**Prior training** (*n* = 31)	**No prior training** (*n* = 12)	***χ*2**	***p*-value**
**Encourage to cough**				1.601	0.206
Yes	21 (48.8)	17 (54.8)	4 (33.3)		
No	22 (51.2)	14 (45.2)	8 (66.7)		
**Give 5 back blows**				12.429	< 0.001
Yes	31 (71.1)	27 (87.1)	4 (33.3)		
No	12 (27.9)	4 (12.9)	8 (66.7)		
**Give black blows correctly**	*n* = 31	*n* = 27	*n* = 4	0.005	0.945
Yes	16 (51.6)	14 (51.9)	2 (50.0)		
No	15 (48.4)	13 (48.1)	2 (50.0)		
**Give 5 abdominal thrusts**				0.509	0.476
Yes	41 (95.3)	30 (96.8)	11 (91.7)		
No	2 (4.7)	1 (3.2)	1 (8.3)		
**Give abdominal thrusts correctly**	*n* = 41	*n* = 30	*n* = 11	3.095	0.079
Yes	7 (17.1)	7 (23.3)	0 (0.0)		
No	34 (82.9)	23 (76.7)	11 (100.0)		
**Continue to 5 back blows and 5 abdominal thrusts**				3,411	0.065
Yes	24 (55.8)	20 (64.5)	4 (33.3)		
No	19 (44.2)	11 (35.5)	8 (66.7)		
**Continue to 5 back blows and 5 abdominal thrusts correctly**	*n* = 24	*n* = 20	*n* = 4	0.960	0.327
Yes	4 (16.7)	4 (20.0)	0		
No	20 (83.3)	16 (80.0)	4 (100.0)		
**Start BLS for unconscious victim**				1.856	0.173
Yes	25 (58.1)	20 (64.5)	5 (41.7)		
No	18 (41.9)	11 (35.5)	7 (58.3)		
**Perform all the steps**				3.237	0.072
Yes	7 (16.3)	7 (22.6)	0 (0.0)		
No	36 (83.7)	24 (77.4)	12 (100.0)		
**Correct compliance rate**^**a**^	50.0 (37.5 – 62.5)	50.0 (50.0–62.5)	31.3 (12.5–50.0)	-	U = 76,000; *p* = 0.002^b^
**Time to back blows (sec)**	13.2 (10.3 – 19.0)	13.5 (10.5–19.2)	9.4 (6.3–13.2)	-	U = 26,000; *p* = 0.107^b^
**Time to abdominal thrust (sec)**	22.5 (14.2 – 29.1)	26.1 (19.2–31.0)	14.1 (9.5–19.8)	-	U = 58,000; *p* = 0.001^b^
**Total time (sec)**	54.4 (42.9 – 67.6)	56.5 (51.9–71.0)	42.7 (40.7–51.2)	-	U = 77,000; *p* = 0.002^b^

Continuous variables are expressed with median (interquartile range)

Categorical variables are expressed with absolute frequency (relative frequency)

*FBAO*  Foreign Body Airway Obstruction, *BLS* Basic Life Support, *sec* seconds, *U* Mann–Whitney U test value

*p*-values calculated by Chi-square test

^a^Score calculated according to correct or incorrect execution of the eight steps of the sequence

^b^Mann-Whitney U test

Concerning the steps of the current recommended protocol, less than 50% of the participants encouraged the victim to cough. Although 71.1% of the participants performed interscapular clapping and 95.3% performed abdominal thrusts, only 51.6% and 17.1% were performed correctly, respectively. Almost all participants who failed to perform the back blows and the abdominal thrusts did so by administering an incorrect number of them. Although 55.8% of the participants continued with the 5 black blows × 5 abdominal thrust sequence, it was only correctly performed in 16.7% of the tests, in most cases by executing abdominal thrusts only (Table 4).

Finally, participants were asked about their opinion on which anti-choking device they would choose after having used both of them, 55.8% of the participants chose the LifeVac® device.

## Discussion

In our study, we have tried to evaluate, in a simulated FBAO scenario, the handling of brand-new anti-choking devices by health science students, as well as observe how they would solve the event following the recommended protocol. In general, the participants have shown greater ease in performing the skills required in the use of anti-choking devices than in handling the currently FBAO recommended protocol.

Although these devices are not yet recommended by resuscitation guidelines [1], they are available to everyone in public places such as airports, shopping centers or schools. Among the scarce and low-quality scientific evidence available to date, there are only two studies that compare both anti-choking devices [20, 21]. The remaining studies, despite reporting a high success rate of airway clearance, only independently evaluate either the LifeVac® device [8–11, 14, 22–24] or the DeCHOKER® device [25]. Besides, only a few studies are case series [8, 9, 23, 25], albeit with a very small sample (n ≤ 29). The rest [11–14, 22, 24], are simulation studies on manikins, except for Juliano et al. [14] who conducted a study on cadavers. In our study, we did not evaluate efficacy but the correct execution technique and times in the different tests. We consider it necessary to evaluate first the knowledge and practical skills in the resolution of the FBAO event both with the devices and with the recommended protocol, before assessing their efficacy in terms of successful foreign body removal.

In general, we observed a correct execution of all steps recommended by the manufacturers in both devices and no differences between them. The most failed step was, also in Carballo-Fazanes et al. [20] study, “*to keep the mask fixed to the victim’s airway throughout the procedure*” although more than 70% of the participants achieved this with both devices. This is reflected in a high estimated correct compliance rate with both devices, being slightly higher in the case of the DeCHOKER®. Regarding the overall time of each scenario was significantly longer with DeCHOKER® than with LifeVac®, as well as Carballo-Fazanes et al. [20] study. These differences, based on our findings and the participants’ comments, may be related to the relative clarity of the LifeVac® instructions for use. In this regard, our results are in line with those obtained by Patterson et al. [21], they observed a significantly higher success rate of FBAO removal in less than 60 sec. with LifeVac® than with DeCHOKER® (82.2% and 44.4% respectively).

By contrast, the current recommended protocol of action for FBAO treatment turned out to be less well known by health science students. The most unknown items of the protocol for our participants were to encourage the victim to cough and the number of back blows and abdominal thrusts. Related to this, we obtained a correct compliance rate of 50%, this rate drops to 31.3% in the case of participants with no prior training. We hypothesise that this could be related to the fact that, despite that some of the participants reported previous training and they are health science students, the current recommended protocol has more steps than anti-choking devices procedures. In addition, anti-choking device tests (which are prepared for laypersons, according to their manufacturers) were carried out following the manufacturer's leaflet instructions, while recommended protocol test was performed without instructions, following their knowledge to solve it.

In this sense, Patterson et al. [21] observed a success rate of FBAO removal of 66.7% in less than 60 sec. with the abdominal thrust procedure. This could also mean that performing the recommended protocol properly is more difficult than using the devices. However, in their study, they compared the efficacy and usefulness of both devices (LifeVac® and DeCHOKER®) with only the abdominal thrusts step of the recommended protocol. Also, more participants reported prior training in BLS (94.4%) than in our study (72.1%). However, we cannot assume that students are well trained in this field.

This study has not tested whether pre-test training would contribute to better results, since we wanted to analyze the first impression and performance with the devices without prior knowledge. However, we hypothesise that this might be the same thing as with other basic life support content, such as automated external defibrillators [26] or adult and child CPR [27–29], where brief pre-test training showed better results.

We asked participants to give their subjective opinion of the devices once they had completed the tests, more than 50% preferred LifeVac®. Although they found DeCHOKER® to be safer, LifeVac® resulted in more intuitive, and practical and had clearer instructions. In previous studies, LifeVac® was also considered superior in terms of ease of use, safety, and confidence by participants [21].

Simulation-based assessment was applied in this study considering its benefits in the emergency medicine setting, as it allows the opportunity to practice clinical skills in a risk-free environment, acquiring special relevance in health science students as it helps to gain self-confidence and willingness in their performance in real clinical settings [30–32].

Our study has some limitations to be considered. First, the relatively small and convenience sample and the imbalance between the number of participants with and without prior training in FBAO protocol. COVID-19 pandemic restrictions have made it difficult to recruit a bigger sample, so our findings may be difficult to generalize. Second, the use of a manikin model in a simulated scenario may not directly translate results to real FBAO events. In this sense, participants may act differently in real-life situations since simulations, especially those involving rapid intervention, often have a Hawthorne effect involving changes in their behaviour because they know they are being observed. On the other hand, a CPR manikin was used (FBAO non-specific) since there is currently no manikin prepared to use the anti-choking devices. The three scenarios (LifeVac® test, DeCHOKER® test, and recommended protocol test) were not compared because the correct execution of the recommended protocol technique could not be evaluated with the commercially available manikin. Therefore, recommended protocol test was performed on a human victim. Finally, no washout period has been established between the scenarios due to the existing differences among them; however, the start of the tests has been randomized so that, in case of a possible learning bias, they are equally affected.

## Conclusions

Untrained Medicine and Nursing students are able to quickly and adequately use the brand-new anti-choking devices (LifeVac® and DeCHOKER®) but fail to apply the current recommended FBAO protocol. This must be considered to define the role of such devices in the case of FBAO and to train future healthcare professionals to adequately manage FBAO events.

## Data Availability

The datasets used and/or analysed during the current study are available from the corresponding author on reasonable request.

## Abbreviations

BLS: Basic Life Support
CPR: Cardiopulmonary Resuscitation
FBAO: Foreign Body Airway Obstruction
IQR: Interquartile Range
